# Global prevalence and burden of meal-related abdominal pain

**DOI:** 10.1186/s12916-022-02259-7

**Published:** 2022-02-17

**Authors:** Esther Colomier, Chloé Melchior, Joost P. Algera, Jóhann P. Hreinsson, Stine Störsrud, Hans Törnblom, Lukas Van Oudenhove, Olafur S. Palsson, Shrikant I. Bangdiwala, Ami D. Sperber, Jan Tack, Magnus Simrén

**Affiliations:** 1 Department of Molecular and Clinical Medicine, Institute of Medicine, Sahlgrenska Academy, University of Gothenburg, Gothenburg, Sweden; 2 Translational Research Center for Gastrointestinal Disorders (TARGID), Department of Chronic Diseases and Metabolism (CHROMETA), KU Leuven, Leuven, Belgium; 3 INSERM UMR 1073, Institute for Research and Innovation in Biomedicine, Normandy University, Rouen, France; 4 Rouen University Hospital, Gastroenterology Department and INSERM CIC-CRB 1404, F-76031 Rouen, France; 5 Laboratory for Brain-Gut Axis Studies (LaBGAS), Translational Research Center for GI Disorders (TARGID), KU Leuven, Leuven, Belgium; 6 Cognitive & Affective Neuroscience Lab (CANlab), Department of Psychological and Brain Sciences, Dartmouth College, Hanover, NH USA; 7 Center for Functional GI and Motility Disorders, University of North Carolina-Chapel Hill, Chapel Hill, NC USA; 8 Department of Health Research Methods, Evidence and Impact, McMaster University, Hamilton, Ontario Canada; 9 Population Health Research Institute, McMaster University, Hamilton, Ontario Canada; 10 Faculty of Health Sciences, Ben-Gurion University of the Negev, Beer-Sheva, Israel

**Keywords:** Meal-related abdominal pain, Global prevalence, Burden, Gastrointestinal symptoms, Disorders of the gut-brain interaction, Functional gastrointestinal disorders, Epidemiology, Food

## Abstract

**Background:**

Patients with disorders of gut-brain interaction (DGBI) report meal intake to be associated with symptoms. DGBI patients with meal-related symptoms may have more severe symptoms overall and worse health outcomes, but this subgroup has not been well characterized. We aimed to describe the global prevalence of meal-related abdominal pain and characterize this subgroup.

**Methods:**

The data analyzed originated from the Internet survey component of the population-based Rome Foundation Global Epidemiology Study, completed in 26 countries (*n* = 54,127). Adult subjects were asked whether they had abdominal pain and how often this was meal-related. Respondents were categorized into “no,” “occasional,” and “frequent” meal-related abdominal pain groups based on 0%, 10–40%, and ≥50% of the pain episodes being meal-related, respectively. DGBI diagnoses, frequency of other GI symptoms, psychological distress, non-GI somatic symptoms, quality of life, and healthcare utilization were compared between groups. Mixed linear and ordinal regression was used to assess independent associations between psychological distress, non-GI somatic symptoms, quality of life, other GI symptoms, and meal-related abdominal pain.

**Results:**

Overall, 51.9% of the respondents reported abdominal pain in the last 3 months, and 11.0% belonged to the group with frequent meal-related abdominal pain, which included more females and younger subjects. DGBI diagnoses were more common in subjects with frequent meal-related abdominal pain, and the frequency of several GI symptoms was associated with having more frequent meal-related abdominal pain. Having meal-related abdominal pain more frequently was also associated with more severe psychological distress, non-GI somatic symptoms, and a poorer quality of life. The group with frequent meal-related abdominal pain also more often consulted a doctor for bowel problems compared to the other groups of meal-related abdominal pain.

**Conclusion:**

Reporting frequent meal-related abdominal pain is common across the globe and associated with other GI and non-GI somatic symptoms, psychological distress, healthcare utilization, and a poorer quality of life. Individuals who frequently experience meal-related abdominal pain also more frequently fulfill the diagnostic criteria for DGBI. Assessing meal-related symptoms in all DGBI patients could be of major importance to improve and individualize symptom management.

**Supplementary Information:**

The online version contains supplementary material available at 10.1186/s12916-022-02259-7.

## Background

Disorders of gut-brain interaction (DGBI) are diagnosed by a typical gastrointestinal (GI) symptom pattern, in the absence of alarm features or evidence of organic disease processes that can account for the symptoms, and after a minimal relevant clinical evaluation [1]. According to the worldwide epidemiology study by Sperber et al., more than 40% of the global adult population can be diagnosed with a DGBI using the Rome IV criteria [2]. It is therefore logical that these disorders are one of the leading causes for consultations in primary care [3, 4]. They negatively influence the quality of life of patients and have a high impact on healthcare costs [5–7].

The pathophysiology of DGBI is multifactorial and, to date, has remained only partly understood. Patients may have or experience motility disturbances, visceral hypersensitivity, altered gut microbiota, altered immune and mucosal functions, and psychosocial disturbances together with a disrupted interaction between the gut and the brain [1]. More recently, food intolerance has been recognized as an important pathophysiological factor [8, 9].

Currently, there are no objective biomarkers available for DGBI. Therefore, diagnosing a patient with a DGBI can be done using the specific Rome IV criteria assessed with the Rome IV questionnaire [10]. These criteria are based on clusters of symptoms derived from a factor analysis after ruling out an identifiable organic disease. The Rome Foundation has been drafting and updating these criteria for more than two decades. The organization characterizes DGBI based upon the anatomical subdivisions in the GI tract. The diagnostic criteria for several DGBI, including functional dysphagia, postprandial distress syndrome, and rumination syndrome, comprise meal-related symptoms [9]. The diagnostic criteria for irritable bowel syndrome (IBS) do not include the presence of meal-related symptoms, although many patients with IBS report an association between food intake and symptom onset [11, 12]. Furthermore, a recent study indicated that 13% of the patients with IBS severely self-restrict and avoid certain foods [13]. These IBS patients appear to represent a more severely affected patient group with a lower quality of life, a reduced caloric intake, and a lower intake of several nutrients. Thus, there are indications that DGBI patients who have meal-related symptoms constitute a distinct subgroup with specific symptom patterns, health outcomes, and pathophysiological alterations.

Although the meal-related factor remains incompletely understood, foods containing poorly absorbed short-chain carbohydrates, i.e., fermentable oligo-, di-, monosaccharides, and polyols (FODMAPs), fatty foods, coffee, alcohol, and hot spices have been identified as the most important dietary symptom triggers [14]. Various diets are increasingly implemented as treatment options not only for patients with IBS, but also for patients with functional dyspepsia [15–18]. Between studies, efficacy rates of dietary interventions generally vary from 50 to 75%, indicating that only a subset of patients responds to the dietary treatment [18–21]. Unfortunately, the specific subgroup of DGBI patients suffering from meal-related symptoms who potentially benefit from dietary intervention is currently not sufficiently characterized. This makes identification of these patients and effectively relieving their symptoms with dietary interventions even more difficult.

Our aim for this study was to describe the global prevalence of meal-related abdominal pain. Furthermore, to characterize subjects with meal-related abdominal pain, we aimed to describe the association between the frequency of meal-related abdominal pain and other GI symptoms, DGBI diagnoses, psychological distress, quality of life, and healthcare utilization. We hypothesized that subjects who frequently experience meal-related abdominal pain have a more severe symptom pattern, a poorer quality of life, and a higher healthcare utilization.

## Methods

### Participants

Participants were originally recruited for the Rome Foundation Global Epidemiology Study, previously described in detail elsewhere [2, 22]. Initially, this study was set up to investigate the global prevalence of DGBI using household and Internet survey data originating from 33 countries worldwide. All subjects who completed the Internet survey of the epidemiology study were included in this particular analysis. For the Internet survey, Qualtrics (Provo, UT, USA), which is a global market survey company, was commissioned to provide a nationally representative general population sample in 26 countries (Argentina, Australia, Belgium, Brazil, Canada, China, Colombia, Egypt, France, Germany, The Netherlands, Israel, Italy, Japan, Mexico, Poland, Romania, Russia, Singapore, South Africa, South Korea, Spain, Sweden, Turkey, UK, and the USA). The individuals who were recruited to complete the survey had previously registered themselves to participate in online surveys, such as opinion polls and health studies. To avoid selection bias, the individuals were not informed that the purpose of the study was specifically to assess the prevalence of DGBI and GI symptoms, before giving electronic consent. Instead, the survey was broadly described as a health survey. The participants remained anonymous to the investigators throughout the process. Further information about the quota-based sampling method that was used and about the study population in general can be found elsewhere [2].

Within the study sample, we aimed to identify and characterize subjects with meal-related abdominal pain. We identified participants who reported experiencing abdominal pain by selecting respondents who did not answer “Never” on the question “In the last 3 months, how often did you experience pain anywhere in your abdomen?” The GI symptom assessed in this question is referred to here as “general abdominal pain.” Among the subjects who reported general abdominal pain, we focused on how frequently the general abdominal pain was meal-related. Participants reporting general abdominal pain were asked a second question: “How often did your pain start or get worse after eating a meal?” Answers were scored from 0% of the time with pain to 100% of the time with pain on an 11-point scale (10% steps, with no response options in between). Respondents were then categorized into three subgroups of meal-related abdominal pain: the “no” meal-related abdominal pain group (0% of the abdominal pain episodes were meal-related), the “occasional” meal-related abdominal pain group (10–40% of the abdominal pain episodes were meal-related), and the “frequent” meal-related abdominal pain group (≥50% of the abdominal pain episodes were meal-related). We used these specific cutoffs of the frequency of meal-related abdominal pain to make the variable more comprehendible, which makes the interpretation of results more relatable to daily life.

### Questionnaires

#### DGBI diagnoses

The Adult Rome IV Diagnostic Questionnaire and a self-reported checklist of organic diseases and surgeries that can cause GI symptoms were used to diagnose DGBI in the global study population. Participants with a self-reported history that could represent organic or structural reasons for their symptoms were excluded from all the Rome IV DGBI diagnoses. Subjects with a history of peptic ulcer disease were excluded from the esophageal, gastroduodenal, and biliary diagnoses; participants who reported diverticulitis or bowel resection were excluded from the bowel and anorectal diagnoses; and subjects reporting celiac disease, GI cancer, or inflammatory bowel disease (Crohn’s disease or ulcerative colitis) were excluded from all Rome IV DGBI diagnoses.

#### GI symptoms

Besides using the Adult Rome IV Diagnostic Questionnaire for the validated assessment of DGBI diagnoses, the questionnaire was used to measure the frequency of individual GI symptoms. Apart from general abdominal pain and meal-related abdominal pain, the frequency of the following other GI symptoms were included in the analysis: sensation of a lump in the throat, retrosternal chest pain, heartburn, dysphagia, postprandial fullness, early satiety, epigastric pain and burning, nausea, vomiting, regurgitation, belching, bloating or abdominal distention, biliary pain, accidental leakage of stool, aching, pain or pressure in the rectum not associated with a bowel movement, hard/lumpy stool, <3 stools per week (without laxative medication or enema), stool straining, and feeling of incomplete emptying. These symptoms were assessed using one-item questions that were structured as follows: “In the last 3 months, how often did you have a specific symptom?” The answers were either reported on a 9-point Likert scale from zero (never) to eight (multiple times per day or all the time) or on an 11-point Likert scale from 0% of times or instances to 100% of the times or instances, in 10% steps.

#### Non-GI symptoms

Non-GI somatic symptoms were measured using the Patient Health Questionnaire (PHQ)-12, which is a modified version of the PHQ-15 excluding the three GI-related questions [23]. Symptoms were measured on a scale from zero = “not bothered at all” to two = “bothered a lot” with a 2-week recall period. The total score (0–24) is created by summing up the separate symptom scores, with high sum scores representing a high non-GI somatic symptom burden. One item assessed menstrual cramps or other period problems. Therefore, women could generally have higher PHQ-12 sum scores.

Psychological distress was assessed using the PHQ-4 questionnaire which is a 4-item questionnaire measuring the amount of anxiety and depression symptoms in the past 2 weeks [24]. Outcomes are scores ranging from zero = “not at all” to three = “nearly every day” that can be summed up to calculate an anxiety, a depression, and a psychological distress score. Higher sum scores represent more severe indications of psychological distress.

#### Quality of life

The Patient-Reported Outcomes Measurement Information System Global-10 (PROMIS Global-10) questionnaire was used to assess the general quality of life of the participants. Nine general statements about subjective quality of life, social, physical, and mental functioning and fatigue are rated by respondents on 5-point Likert scales, and the tenth question, about overall pain, on an 11-point Likert scale. The results are two total scores, one for physical and one for mental quality of life with high scores representing a good quality of life [25].

#### Healthcare utilization

The study survey contained questions measuring healthcare utilization, medication use, and surgery history. One question that was used in our analyses assessed if the subjects had ever visited a doctor because of a bowel problem. The second question assessed what particular doctor the subjects consulted for a bowel problem. Possible answers included a general practitioner or family doctor, a gastroenterologist, a gynecologist, a surgeon, a folk healer or traditional healer, an ayurvedic doctor, a homeopathic doctor, a traditional Chinese medicine doctor, and a chiropractor.

### Data analysis

Prevalence rates were pooled across countries to determine a general prevalence rate. For this purpose, Yang’s meta-prevalence method, which combines separate population survey prevalence estimates into an overall meta-prevalence estimate, was used [26].

Proportions and means with 95% confidence intervals (95% CIs) were calculated for the three groups of meal-related abdominal pain in tables and graphs. Hereby, we focused on demographic variables, DGBI diagnoses, healthcare utilization, and the frequency of other GI symptoms. Among the subjects from the three groups of meal-related abdominal pain, we also investigated the proportion of subjects who fulfill the diagnostic criteria for zero, one, two, three, or four DGBI. Within the group fulfilling zero, one, two, three, or four DGBI diagnoses, we calculated the proportion of subjects from the no, occasional, and frequent meal-related abdominal pain group. To describe and summarize the data in this manner, no statistical tests were performed.

To be able to draw conclusions from the collected data, mixed ordinal regression was then used to analyze the association between meal-related abdominal pain (dependent variable) and the frequency of other GI symptoms (a priori chosen independent variables of interest). Potential confounders that were added to the models included demographical variables (age, gender, education, BMI) and psychological distress. A base model with the confounding variables was constructed and each GI symptom frequency was then individually added to the base model (Additional file 1: Table S1). Country was included as a random intercept effect to account for variability among countries. The intraclass correlation coefficient (ICC), when only including meal-related abdominal pain as the dependent variable and country as random intercept, was 0.014. The estimates of effect sizes of the independent variables were reported as odds ratios (ORs) and their 95% CIs. To give an example for interpretation of the ORs in ordinal regression, an *OR* of 1.4 for the continuous variable nausea (measured on a 11-point scale) means that the odds of having more frequent meal-related pain were increased by 1.4 for every unit increase in nausea. The proportional odds assumption for ordinal regression was in many cases not met. However, it was used for the analysis nonetheless as linear regression, beta regression, zero-inflated beta regression, and zero-inflated negative binomial regression fitted the data poorly. This was evaluated with diagnostic plots of residuals using the “DHARMa” package [27] in R”. The package “ordinal” [26] was used for the mixed ordinal regression analysis and the code can be provided upon request.

In order to assess the association between meal-related abdominal pain and health outcomes such as non-GI somatic symptoms, psychological distress, and quality of life, mixed linear regression was used. Meal-related abdominal pain was added as an independent variable and the dependent variables included psychological distress, non-GI somatic symptoms, and physical and mental quality of life (each included separately in the models). Potential confounders were considered the same as described for the ordinal regression. Country was included as random intercept effect.

Variance inflation factors were used to assess multicollinearity which were low (<5) in all cases.

Analyses were carried out with R (version 4.1.1) and R Studio (version 1.3.1093).

## Results

### Worldwide prevalence of frequent meal-related abdominal pain

**Fig. 1:**
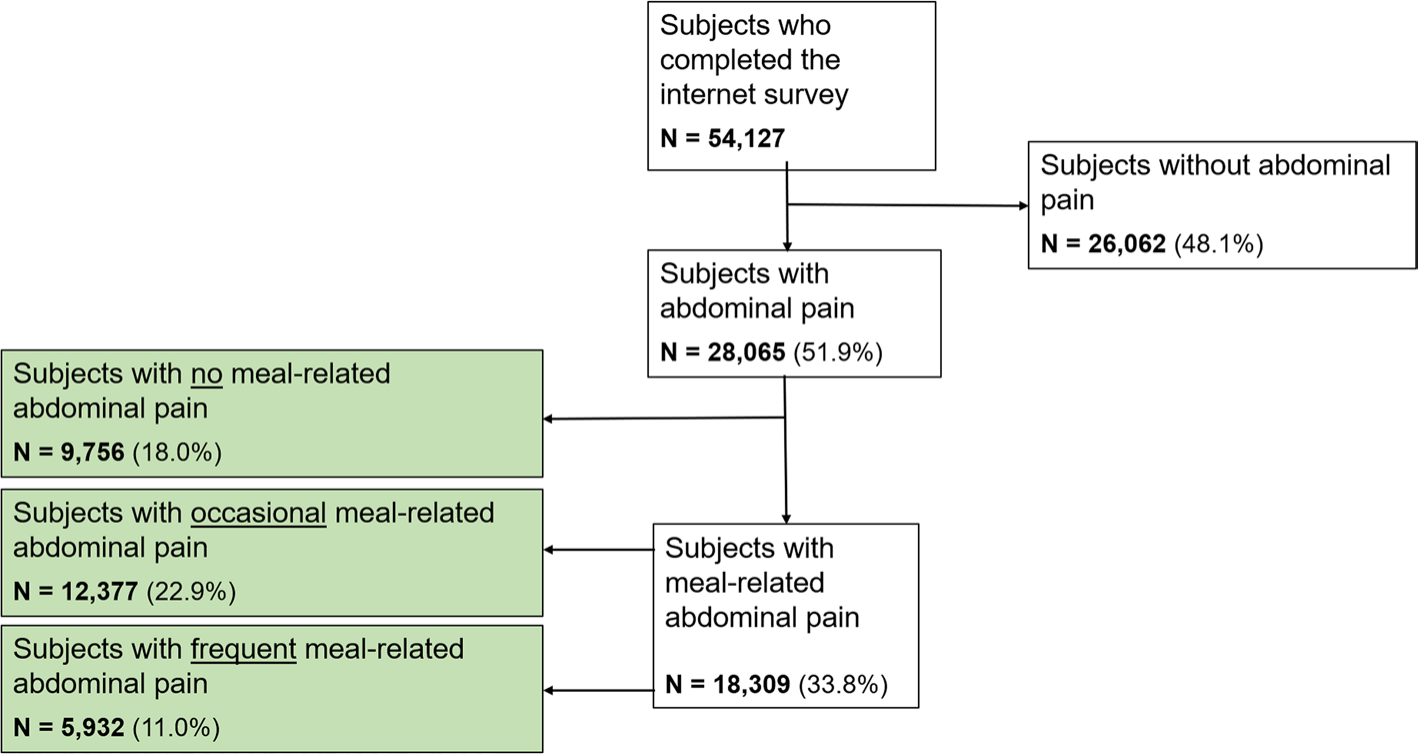
Flowchart of the subjects participating and reporting meal-related abdominal pain. We included subjects who completed the internet-based survey of the Rome Foundation Global Epidemiology Study. The analysis focused on individuals who report abdominal pain that is related to meal intake. If subjects reported meal-related abdominal pain, they were categorized into three subgroups: subjects reporting no (0% of the abdominal pain episodes were meal-related), occasional (10–40% of the abdominal pain episodes were meal-related), and frequent (≥ 50% of the abdominal pain episodes were meal-related) meal-related pain.

**Fig. 2:**
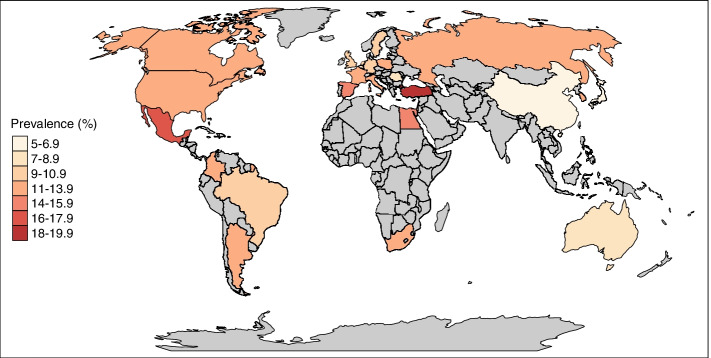
The prevalence of frequent meal-related abdominal pain across the participating countries. The global prevalence of frequent meal-related pain (≥ 50% of the abdominal pain episodes were meal-related) was determined in the adult population and differed across countries, ranging from 5.1% in Italy to 18.0% in Turkey. A total of 54,127 subjects were included in the study of which 5932 experienced frequent meal-related abdominal pain (11.0%). The countries colored in gray did not participate in the Rome Foundation Global Epidemiology Study

As displayed in the flowchart, we found that 51.9% of the global population reported that they experienced abdominal pain and 48.1% never experienced abdominal pain in the last 3 months (Fig. 1). In total, 18.0% of the global population reported that if they experienced abdominal pain, it was never meal-related, 22.9% indicated that they occasionally had meal-related abdominal pain (10–40% of abdominal pain episodes), and 11.0% reported frequent meal-related abdominal pain (at least 50% of abdominal pain episodes). As shown in Fig. 2 and Table 1, differences across countries were apparent. In China, Singapore, and Italy, 5.0–6.9% of the population frequently experienced meal-related abdominal pain, whereas the prevalence rates were clearly higher in Turkey, South Korea, and Egypt (14.0–18.0%). Table 2 shows the demographic characteristics of the three subgroups based on the frequency of meal-related abdominal pain. The highest proportions of females and younger individuals were observed in the group with frequent meal-related abdominal pain, as compared to the other two groups. When looking at the different BMI groups and education levels (as a proxy for socio-economic status), no major differences in proportion rates were found between the groups.

**Table 1:** Proportion of subjects with frequent meal-related abdominal pain within each region

Region	Frequent meal-related abdominal pain (*n* = 5,932)
*Worldwide*	11.0 (10.7, 11.2)
*Africa*	
South Africa	12.0 (10.7, 13.5)
*Asia*	7.1 (6.6, 7.6)
China	6.9 (6.0, 7.8)
Japan	5.3 (4.5, 6.2)
Singapore	5.7 (4.8, 6.8)
South Korea	11.2 (9.9, 12.7)
*Australia*	8.1 (7.0, 9.3)
*Eastern Europe*	11.0 (10.2, 11.8)
Poland	12.8 (11.5, 14.4)
Romania	8.3 (7.1, 9.5)
Russia	11.9 (10.6, 13.4)
*Latin America*	12.5 (11.8, 13.3)
Argentina	12.2 (10.8, 13.6)
Brazil	10.4 (9.2, 11.8)
Colombia	12.3 (10.9, 13.8)
Mexico	15.3 (13.8, 16.9)
*Middle East*	15.0 (14.1, 15.9)
Egypt	14.4 (12.9, 16.0)
Israel	12.6 (11.2, 14.2)
Turkey	18.1 (16.4, 19.8)
*North America*	11.9 (10.9, 12.9)
Canada	12.2 (10.8, 13.7)
USA	11.6 (10.3, 13.0)
*Western Europe*	10.9 (10.4, 11.4)
Belgium	9.0 (7.8, 10.3)
France	11.9 (10.6, 13.4)
Germany	10.9 (9.7, 12.4)
The Netherlands	9.0 (7.8, 10.4)
Italy	12.6 (11.2, 14.1)
Spain	13.5 (12.1, 15.1)
Sweden	10.6 (9.3, 12.0)
UK	9.8 (8.6, 11.1)

The proportion of subjects with “frequent” meal-related abdominal pain was determined within each country and region. “Frequent”: abdominal pain ≥ 50% of the time meal-related. Data are presented as percentage (95% confidence interval)

**Table 2:** Demographic characteristics of subjects grouped by frequency of meal-related abdominal pain

***Demographic***	Frequency of meal-related abdominal pain		
	No (***n*** = 9756)	Occasional (***n*** = 12,377)	Frequent (***n*** = 5932)
*Gender*			
Female	57.2 (56.2, 58.2)	54.6 (53.7, 55.5)	58.8 (57.6, 60.1)
Male	42.8 (41.8, 43.8)	45.4 (44.5, 46.3)	41.2 (57.6, 60.1)
*Age groups (years)*			
18–29	24.8 (23.9, 25.7)	28.2 (27.4, 29.0)	31.1 (29.9, 32.3)
30–44	31.5 (30.6, 32.4)	34.7 (33.9, 35.6)	36.5 (35.2, 37.7)
45–59	23.5 (22.6, 24.3)	22.8 (22.0, 23.5)	21.8 (20.7, 22.9)
60–74	19.0 (18.2, 19.8)	13.4 (12.8, 14.0)	10.2 (9.4, 11.0)
75–100	1.2 (1.0, 1.4)	1.0 (0.8, 1.2)	0.5 (0.4, 0.8)
*BMI groups (kg/m*^*2*^*)*			
Underweight^a^	5.1 (4.6, 5.6)	5.4 (5.0, 5.9)	6.0 (5.4, 6.7)
Normal weight^b^	46.7 (45.6, 47.7)	49.4 (48.5, 50.3)	47.1 (45.8, 48.5)
Overweight^c^	29.9 (29.0, 30.9)	28.6 (27.8, 29.5)	27.7 (26.5, 28.9)
Obese I^d^	11.8 (11.1, 12.5)	10.8 (10.2, 11.4)	12.3 (11.5, 13.3)
Obese II^e^	4.4 (4.0, 4.8)	3.6 (3.3, 4.0)	4.4 (3.8, 5.0)
Obese III^f^	2.1 (1.8, 2.4)	2.2 (1.9, 2.4)	2.4 (2.0, 2.9)
*Education levels (years)*			
0–7	9.6 (9.0, 10.2)	9.9 (9.4, 10.5)	11.1 (10.3, 12.0)
8–14	50.4 (49.4, 51.4)	48.1 (47.2, 49.0)	46.3 (45.0, 47.6)
15–40	40.0 (39.0, 41.0)	42.0 (41.1, 42.9)	42.5 (41.2, 43.8)

The proportion of subjects with a specific demographic characteristic in groups “no,” “occasional,” and “frequent” meal-related abdominal pain was determined within each group. “No”: abdominal pain 0% of the time meal-related; “occasional”: abdominal pain 10–40% of the time meal-related; “Frequent”: abdominal pain ≥ 50% of the time meal-related. Data are presented as percentage (95% confidence interval)

^a^BMI < 18.5 kg/m^2^, ^b^18.5 kg/m^2^ < BMI < 24.9 kg/m^2^, ^c^25 kg/m^2^ < BMI < 29.9 kg/m^2^, ^d^30 kg/m^2^ < BMI < 34.9 kg/m^2^, ^e^35 kg/m^2^ < BMI < 39.9 kg/m^2^, ^f^BMI >40 kg/m^2^

### DGBI diagnoses of subjects grouped according to frequency of meal-related abdominal pain

Subjects with frequent meal-related abdominal pain were more likely to fulfill diagnostic criteria for DGBI than the subjects with no or occasional meal-related abdominal pain. For all DGBI, the proportion of subjects fulfilling DGBI diagnoses was larger in the frequent meal-related abdominal pain group compared to the other two groups (Table 3). Within the bowel disorders, the proportion of patients with frequent meal-related abdominal pain was highest in IBS. Functional dyspepsia (mainly the epigastric pain syndrome) and functional dysphagia were the gastroduodenal and esophageal disorders with the highest proportion of patients reporting frequent meal-related abdominal pain, respectively. For more details, see Table 3.

**Table 3:** DGBI diagnoses in subjects grouped by frequency of meal-related abdominal pain

***Diagnosis***	Frequency of meal-related abdominal pain		
	No (***n*** = 9756)	Occasional (***n*** = 12,377)	Frequent (***n*** = 5932)
*Esophageal disorders*	6.6 (6.1, 7.1)	9.4 (8.9, 9.9)	20.2 (19.1, 21.2)
Functional chest pain	2.3 (2.0, 2.6)	2.1 (1.9, 2.4)	2.7 (2.3, 3.1)
Functional heartburn	0.9 (0.7, 1.1)	1.9 (1.7, 2.2)	6.1 (5.5, 6.7)
Reflux hypersensitivity	0.7 (0.6, 0.9)	1.3 (1.1, 1.5)	5.3 (4.7, 5.9)
Globus	1.1 (0.9, 1.3)	0.8 (0.7, 1.0)	1.1 (0.9, 1.4)
Functional dysphagia	2.6 (2.3, 2.9)	5.2 (4.8, 5.6)	13.7 (12.9, 14.6)
*Gastroduodenal disorders*	9.4 (8.9–10.0)	19.4 (18.7, 20.1)	35.0 (33.8, 36.2)
Functional dyspepsia (*n* = 3834)	5.7 (5.2, 6.2)	12.8 (12.2, 13.4)	28.6 (27.5, 29.8)
Postprandial distress syndrome	86.5 (83.7, 89.4)	80.7 (78.7, 82.6)	79.9 (78.0, 81.8)
Epigastric pain syndrome	21.8 (18.3, 21.2)	36.0 (33.6, 38.3)	56.7 (54.4, 59.1)
Belching disorder	0.7 (0.5, 0.9)	1.5 (1.3, 1.7)	5.2 (4.7, 5.8)
Chronic nausea and vomiting syndrome	0.7 (0.5, 0.8)	1.7 (1.5, 1.9)	4.5 (4.0, 5.0)
Cyclic vomiting syndrome	0.6 (0.5, 0.8)	2.6 (2.3, 2.9)	5.5 (4.9, 6.1)
Cannabinoid hyperemesis syndrome	0.0 (0.0, 0.0)	0.0 (0.0, 0.1)	0.4 (0.2, 0.5)
Rumination syndrome	2.9 (2.6, 3.3)	5.3 (5.0, 5.8)	5.9 (5.3, 6.5)
*Bowel disorders*	36.5 (35.6, 37.5)	51.5 (50.6, 52.4)	69.5 (68.3, 70.6)
Irritable bowel syndrome (*n* = 2616)	2.3 (2.0, 2.6)	7.3 (6.8, 7.8)	25.1 (24.0, 26.2)
IBS-C	30.1 (24.2, 0.365)	32.2 (29.1, 35.3)	31.9 (29.5, 34.3)
IBS-D	27.4 (21.7, 33.7)	27.7 (24.8, 30.8)	29.4 (27.1, 31.8)
IBS-M	33.2 (27.1, 39.7)	32.5 (29.5, 35.7)	34.1 (31.7, 36.6)
IBS-U	9.3 (5.8, 13.9)	7.5 (5.9, 9.5)	4.6 (3.6, 5.8)
Functional constipation	13.7 (13.0, 14.4)	18.0 (17.3, 18.7)	18.1 (17.1, 19.1)
Functional diarrhea	4.7 (4.3, 5.1)	6.4 (5.9, 6.8)	7.8 (7.2, 8.6)
Functional abdominal bloating/distention	4.3 (3.9, 4.7)	5.3 (4.9, 5.7)	5.1 (4.6, 5.7)
Unspecified functional bowel disorder	11.0 (10.4, 11.6)	13.0 (12.5, 13.7)	11.9 (11.1, 12.8)
Opioid-induced constipation	1.1 (0.9, 1.3)	3.5 (3.2, 3.8)	4.7 (4.2, 5.3)
Central abdominal pain syndrome	0.1 (0.0, 0.2)	0.0 (0.0, 0.1)	0.0 (0.0, 0.1)
*Biliary pain*	0.1 (0.1, 0.2)	0.1 (0.1, 0.2)	0.3 (0.2, 0.5)
*Anorectal disorders*	7.4 (6.9, 8.0)	14.3 (13.7, 15.0)	25.2 (24.1, 26.3)
Fecal incontinence	1.1 (0.9, 1.4)	2.8 (2.5, 3.1)	6.4 (5.8, 7.1)
Levator Ani syndrome	1.2 (1.0, 1.4)	1.8 (1.6, 2.1)	5.5 (4.9, 6.1)
Proctalgia Fugax	5.4 (5.0, 5.9)	11.0 (10.4, 11.5)	16.9 (16.0, 17.9)

The proportion of subjects fulfilling the Rome IV criteria for a DGBI in “no” (0% of the abdominal pain episodes were meal-related), “occasional” (10–40% of the abdominal pain episodes were meal-related), and “frequent” (≥ 50% of the abdominal pain episodes were meal-related) group was determined within each group. The rows containing the main DGBI categories indicate the proportion of subjects fulfilling the diagnostic criteria of at least one esophageal, gastroduodenal, bowel, and anorectal disorder. Within the subgroups of functional dyspepsia and irritable bowel syndrome, proportions were calculated based on the total number of functional dyspepsia and irritable bowel syndrome patients, respectively. Data are presented as percentage (95% confidence interval)

*DGBI* Disorder of the gut-brain interaction, *IBS-C* Irritable bowel syndrome with predominant constipation, *IBS-D* Irritable bowel syndrome with predominant diarrhea, *IBS-M* Irritable bowel syndrome with mixed bowel habits, *IBS-U* Irritable bowel syndrome unsubtyped

**Fig. 3:**
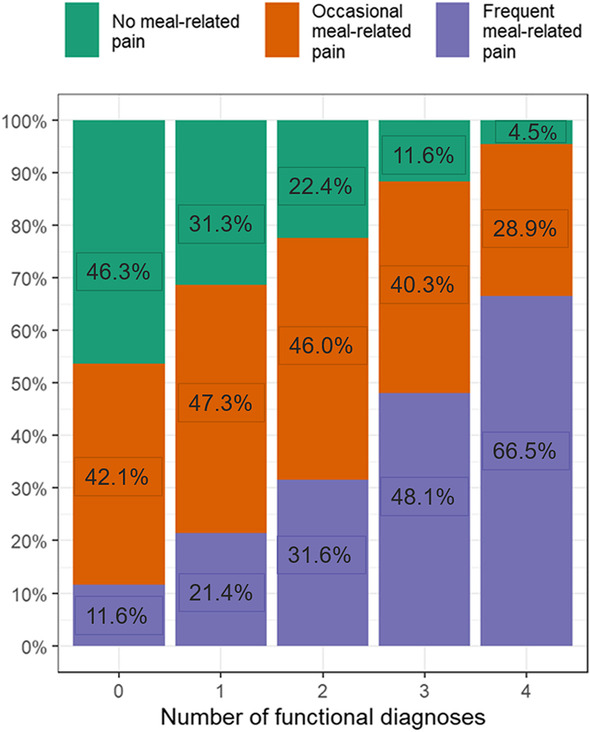
Proportion of subjects having 0–4 DGBI diagnoses grouped according to the frequency of When the number of DGBI diagnoses increased (going from having zero to having four DGBI diagnoses), a gradual increase in the proportion of subjects with frequent meal-related abdominal pain (≥ 50% of the abdominal pain episodes were meal-related) was noted. A gradual decrease in the proportion of subjects with no meal-related abdominal pain (0% of the abdominal pain episodes were meal-related) was observed. DGBI diagnoses were grouped within the main anatomical DGBI categories, i.e., esophageal, gastroduodenal, bowel, and anorectal disorders, for this analysis

With an increasing number of DGBI diagnoses (within the main anatomical DGBI categories), a gradual increase in the proportion of subjects with frequent meal-related abdominal pain was noted (Fig. 3). Within the subgroup of individuals with four DGBI diagnoses, the proportion of subjects with frequent meal-related abdominal pain was higher than the proportion of subjects with occasional and no meal-related abdominal pain (66.5% vs. 28.9% vs. 4.5%, respectively).

### Other GI symptoms associated with meal-related abdominal pain

The GI symptoms that occurred most frequently in all three meal-related abdominal pain groups were bloating or abdominal distention, postprandial fullness, and symptoms of constipation and diarrhea (Additional file 1: Table S2). The largest differences between the groups were seen for bloating or abdominal distention, postprandial fullness, biliary pain, early satiety, epigastric pain and burning, and symptoms of constipation and diarrhea.

**Fig. 4:**
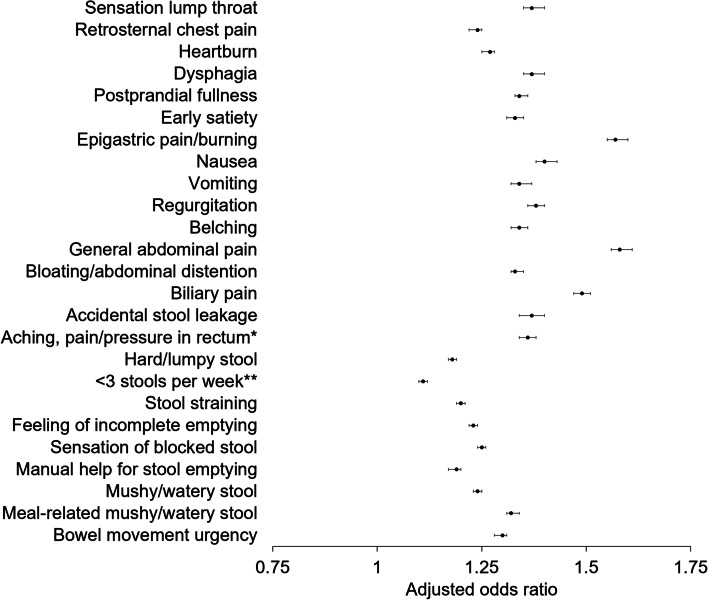
The frequency of having other GI symptoms is associated with having meal-related abdominal pain more frequently. Mixed ordinal regression models with the frequency of meal-related pain (11-item scale, 0–100%) as outcome indicated that having meal-related abdominal pain more frequently was associated with having other GI symptoms more frequently. The frequency of all other GI symptoms questioned in the Adult Diagnostic Rome IV questionnaire was used as an independent variable for the separate mixed ordinal regression models. Country was included as a random intercept effect to account for variability among countries. *OR* > 1 corresponds to higher odds of having meal-related abdominal pain more frequently. All models were corrected for the following confounders: demographical variables (age, gender, education, BMI) and psychological distress. *not associated with a bowel movement. **without laxative medication or enema

In the unadjusted mixed ordinal regression, all of the GI symptoms had a strong association with meal-related abdominal pain (Additional file 1: Fig. S1). Experiencing general abdominal pain (*OR* = 3.77, 95% *CI* = 3.44–4.14), bloating or abdominal distention (*OR* = 2.61, 95% *CI* = 2.38–.87), a feeling of incomplete emptying (*OR*=2.42, 95% *CI* = 2.24–2.62), biliary pain (*OR* = 2.37, 95% *CI* = 2.19–2.57), and postprandial fullness (*OR* = 2.26, 95% *CI* = 2.09–2.46) corresponded to higher odds of having more frequent meal-related abdominal pain. In the adjusted analysis, age, gender, BMI, education (as a proxy for socio-economic status), and psychological distress were considered as possible confounders. After adjustment, the association of GI symptoms and meal-related abdominal pain was still considerable for the variables with the strongest associations seen for general abdominal pain (*OR* = 1.58, 95% *CI* = 1.56–1.61), epigastric pain and burning (*OR* = 1.57, 95% *CI* = 1.55–1.60), biliary pain (*OR* = 1.49, 95% *CI* = 1.47–1.51), nausea (*OR* = 1.40, 95% *C I*= 1.38–1.43), and regurgitation (*OR* = 1.38, 95% *CI* = 1.36–1.40) (Fig. 4).

### Psychological distress, non-GI somatic symptoms, and quality of life associated with meal-related abdominal pain

**Fig. 5:**
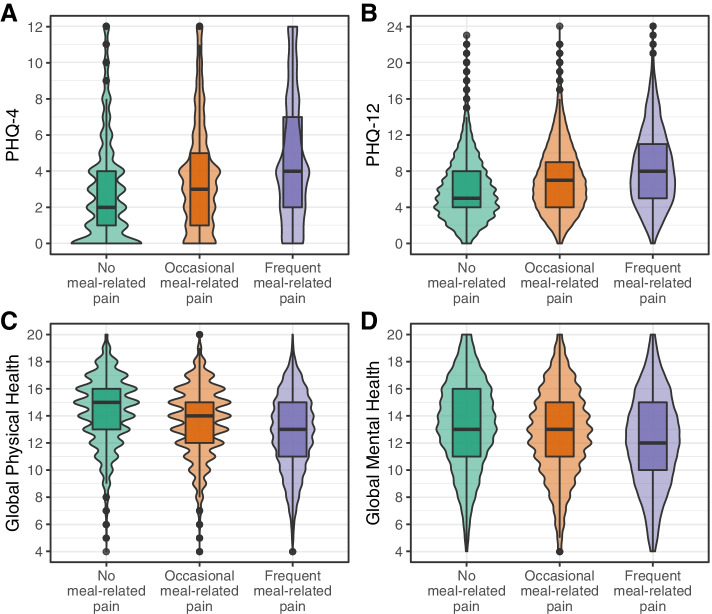
Psychological, somatic conditions and quality of life of subjects grouped by frequency of meal-related abdominal pain. Psychological distress and non-GI somatic symptoms were measured with the PHQ-4 and PHQ-12 questionnaires, respectively. Both measures indicated higher scores in the frequent meal-related abdominal pain group (≥ 50% of the abdominal pain episodes were meal-related). Higher scores represented more severe indications on psychological distress and a higher burden of non-GI somatic symptoms. The physical and mental quality of life of the three groups was assessed with the PROMIS-10 questionnaire. The frequent meal-related abdominal pain group had lower physical and mental quality of life compared to the no (0% of the abdominal pain episodes were meal-related) and occasional (10–40% of the abdominal pain episodes were meal-related) meal-related abdominal pain group

More severe psychological distress was observed in the group with frequent meal-related abdominal pain compared to the other groups (Fig. 5a). Furthermore, the frequent meal-related abdominal pain group showed a higher burden of non-GI somatic symptoms compared to the two other groups (Fig. 5b), as well as a lower physical and mental quality of life (Fig. 5c, d).

The four separate mixed linear regression models indicated that psychological distress, non-GI somatic symptoms, and mental and physical quality of life were all strongly associated with the frequency of meal-related abdominal pain (Additional file 1: Table S3). After adjustment for demographical variables (age, gender, education, BMI) and psychological distress, the strength of the associations decreased for psychological distress, non-GI somatic symptoms, and physical quality of life (Table 4). The association between meal-related abdominal pain and poor mental quality of life diminished when the potential confounders were included in the model.

**Table 4:** Adjusted mixed linear regression

Predictors	Outcome: Psychological distress	Outcome: Non-GI somatic symptoms	Outcome: Physical quality of life	Outcome: Mental quality of life
Intercept	4.21 (3.94, 4.48)	4.15 (3.91, 4.40)	16.68 (16.47, 16.90)	15.80 (15.43, 16.16)
Meal-related abdominal pain	0.22 (0.20, 0.23)	0.22 (0.20, 0.23)	-0.14 (-0.15, -0.13)	-0.02 (-0.03, -0.01)
Age	-0.03 (-0.03, -0.02)	-0.00 (-0.01, -0.00)	-0.03 (0.04, -0.03)	-0.02 (-0.02, -0.02)
Gender (female)	0.57 (0.50, 0.64)	1.42 (1.34, 1.50)	-0.31 (-0.36, -0.26)	-0.13 (-0.18, -0.07)
Education	-0.03 (-0.03, -0.02)	-0.00 (-0.01, 0.01)	0.03 (0.02, 0.03)	0.03 (0.02, 0.04)
Psychological distress	–	0.49 (0.47, 0.50)	-0.41 (-0.42, -0.40)	-0.70 (-0.71, -0.69)

In the adjusted models, the frequency of meal-related abdominal pain had a significant (*p* < 0.05) main effect on all different outcomes. Data are presented as estimates (95% confidence interval)

### Healthcare utilization of subjects grouped according to frequency of meal-related abdominal pain

A higher proportion of subjects with frequent meal-related abdominal pain had consulted a doctor because of a bowel problem. Most subjects in the three groups visited a general practitioner/family doctor or a gastroenterologist for this purpose. Again, the proportion of subjects with frequent meal-related abdominal pain who used this healthcare service was higher than the proportion of subjects in the other two groups (Table 5).

**Table 5:** Healthcare utilization in subjects grouped by frequency of meal-related abdominal pain

***Healthcare utilization***	Frequency of meal-related abdominal pain		
	No (***n*** = 9756)	Occasional (***n*** = 12,377)	Frequent (***n*** = 5932)
Visited doctor because of bowel problems	36.3 (35.3, 37.3)	49.8 (48.9, 50.7)	59.4 (58.2, 60.6)
*Kind of doctor*			
General practitioner or family doctor	28.5 (27.6, 29.4)	36.5 (35.7, 37.3)	46.2 (44.9, 47.5)
Gastroenterologist	17.0 (16.3, 17.7)	24.6 (23.8, 25.4)	30.3 (29.1, 31.5)
Gynecologist	1.9 (1.6, 2.2)	3.1 (2.8, 3.4)	5.3 (4.7, 5.9)
Surgeon	2.9 (2.6, 3.2)	3.8 (3.5, 4.1)	5.2 (4.6, 5.8)
Folk healer or traditional healer	0.3 (0.2, 0.4)	0.6 (0.5, 0.7)	1.2 (0.9, 1.5)
Ayurvedic doctor	0.1 (0.0, 0.1)	0.2 (0.1, 0.3)	0.3 (0.2, 0.4)
Homeopathic doctor	0.9 (0.7, 1.1)	1.5 (1.3, 1.7)	2.5 (2.1, 2.9)
Traditional Chinese medicine doctor	0.6 (0.4, 0.8)	1.5 (1.3, 1.7)	1.8 (1.5, 2.1)
Chiropractor	0.2 (0.1, 0.3)	0.4 (0.3, 0.5)	0.7 (0.5, 0.9)

The proportion of subjects using a specific type of health care in the “no,” “occasional,” and “frequent” groups was determined within each group. “No”: abdominal pain 0% of the time meal-related; “occasional”: abdominal pain 10–40% of the time meal-related; “Frequent”: abdominal pain ≥ 50% of the time meal-related. Data are presented as percentage (95% confidence interval)

## Discussion

To our knowledge, this is the first study to evaluate the global prevalence of frequent meal-related abdominal pain using the Rome IV questionnaire. We show that 11% of the adult global population frequently experiences meal-related abdominal pain. This subgroup is characterized by more females and a younger age. Subjects with frequent meal-related abdominal pain frequently fulfill the diagnostic criteria for several DGBI and more frequently use healthcare services for their bowel symptoms. It is, therefore, not surprising that having meal-related abdominal pain more frequently is independently associated with a higher frequency of having other GI symptoms. Subjects with frequent meal-related abdominal pain also have more severe psychological distress, as well as a higher burden of non-GI symptoms, and a lower physical and mental quality of life.

An overall meta-prevalence of 11% emphasizes that a substantial proportion of the global population experiences abdominal pain associated with meal intake. In this study, we focus on a meal-related GI symptom assessed by a single question. Comparing this 11% to a global prevalence rate of 40% for any DGBI would not be appropriate [2]. However, it does indicate that even though not all individuals who experience meal-related abdominal pain fulfill the diagnostic criteria for a DGBI, a substantial proportion of the global population experiencing this meal-related symptom might need a suitable treatment approach.

The prevalence rates of meal-related abdominal pain ranged from 5.3% in Italy to 18.1% in Turkey. Previous research has shown that the global prevalence rates of DGBI in general vary widely across countries [2, 28–30]. In meta-analyses, differences in prevalence rates across countries and studies can be explained by methodological variances, such as using different diagnostic criteria [3, 29]. By performing this international multicenter study, the heterogeneity in methodology used between study centers was eliminated. However, differences in prevalence rates could still occur as a result of sociocultural factors [31–33]. When assessing meal-related abdominal pain, subjects’ perception of (abdominal) pain undoubtedly plays a role. Patients have symptom-related beliefs that influence how they handle the symptom experience and how they report symptoms [34, 35]. In addition, patients receive different healthcare services across the globe which also affects symptom-related beliefs [36]. Furthermore, different cultural subgroups have different levels of literacy skills which could lead to difficulties understanding the study survey for some individuals [37]. Linguistic differences across countries also occur yielding a difference in the interpretation and reporting of symptoms [38]. Even in the same language, the same word can have a different meaning to different people. In India, people consume a fiber-rich diet and commonly have at least two to three bowel movements per day. When individuals consume a more Westernized diet, they could have only one bowel movement per day which might be interpreted and reported as constipation [39]. Moreover, well-established Rome IV DGBI symptom patterns could differ between patients across the world. In China and India, the majority of the patients with IBS, defined as lower abdominal pain with altered bowel habits in Rome IV, report upper abdominal symptoms, such as bloating, dyspepsia, and epigastric pain relieved by defecation as their key symptom [40]. Apart from the interpretation of symptoms, both dietary-related beliefs and dietary habits are of equal importance. We encounter different diets across the globe including the Western, Asian, Mediterranean, typical Australian diet, and many more. The Western dietary pattern contains a lot of ultra-processed foods, which are associated with symptom severity in patients with IBS and in turn most probably the prevalence of meal-related GI symptoms [41]. In China, diet is believed to be crucial for a person’s health [42]. According to the ancient Chinese wisdom, foods can be categorized into cold and hot foods. Both Chinese and Hispanics believe that diseases can be caused by an imbalance between cold and hot principles that are not related to temperature [43]. Lastly, how we identify food as a potential symptom trigger is of importance. Dietary triggers may not always occur in the form of a meal. In the Adult Rome IV Diagnostic Questionnaire, specific food items, or substrates are not assessed as potential triggering factors. Consequently, we have to consider the potential differences in pathophysiological mechanisms behind meal- and food-related abdominal pain. Many specific food items or substrates, such as FODMAP-rich foods, spicy foods, and specific food antigens, i.e., wheat, yeast, milk, and soy, have been shown to exacerbate symptoms through many different pathophysiological mechanisms, such as incomplete absorption in the small bowel leading to water retention and gas formation in the large intestine, histamine-mediated responses, and local-immune reactions [8, 14, 44, 45]. Meal-related abdominal pain could very well be explained by similar mechanisms, but may also be linked to the caloric load of a meal and alterations in the expected GI motor response after meal intake. Therefore, how the trigger factor is defined in the assessment can influence outcomes. Hence, several factors related to the perception of symptoms, differences in diet, and views on food across the globe can potentially explain regional differences in frequency of meal-related abdominal pain.

When we study the characteristics of subjects who report frequently experiencing meal-related abdominal pain, we observe that more females and younger individuals belong to this subgroup. The majority of DGBI are more prevalent in females than in males with a female/male ratio of approximately 2:1 [3, 46]. Studies propose that the causes of these differences in prevalence rate between males and females are multifactorial and result from psychological [47, 48], social [49, 50], and biological factors, such as GI motility, [51, 52] autonomic tone [53], and central processing of visceral stimuli [54–56]. In regard to age and GI symptoms, most studies report that the prevalence of the majority of DGBI decreases with age [2]. In addition, dietary patterns tend to differ between age groups, e.g., children and adolescents tend to have a higher intake of ultra-processed foods and fructose [57, 58]. We hypothesize that subjects who report frequently experiencing individual GI symptoms reflect similar demographic characteristics as individuals who fulfill diagnostic criteria for DGBI.

The proportion of the subjects in the frequent meal-related abdominal pain group who fulfill the diagnostic criteria for any DGBI was always higher compared to the other two groups. This could indicate that individuals with frequent meal-related abdominal pain represent a patient group with a more severe and diverse symptom pattern. A large proportion of the subjects who report frequently experiencing meal-related abdominal pain fulfill the diagnostic criteria for IBS. This finding was not unexpected since having abdominal pain (associated with altered bowel habits) is a required diagnostic criterion for IBS. However, we observe that a large proportion of subjects with frequent meal-related abdominal pain also fulfill the criteria for other DGBI, mainly DGBI that include other aspects of pain located in different parts of the body (e.g., epigastric pain syndrome and biliary pain) or other meal-related GI symptoms. It is perhaps not a surprise that having one type of pain comes with increased odds of other aspects of pain. However, it is still useful information to report when we aimed to characterize the symptom pattern and health outcomes of individuals who report meal-related abdominal pain. We observe a similar pattern in the co-existing symptoms of individuals with frequent meal-related abdominal pain. The symptoms that were strongly associated with meal-related abdominal pain included either a symptom aspect of pain or imply other symptoms that are related to food intake. Moreover, it has been shown that patients with overlapping DGBI and symptoms have increased disease severity, poorer quality of life, and increased use of healthcare services [22, 59]. We hypothesize that a large proportion of the individuals from the group with frequent meal-related abdominal pain represents this specific patient population with more severe health outcomes and diverse symptom patterns. When we look at the health outcomes in our analysis, a higher burden of psychological distress, more severe non-GI somatic symptoms, and a reduced quality of life were all independently associated with having meal-related abdominal pain more frequently. This might again be explained by the fact that patients with frequent meal-related abdominal pain experience a more severe symptom pattern that emphasizes the central role of the gut-brain axis in DGBI. The substantial decrease in effect size we noticed for the association with mental quality of life could potentially be explained by the effect of one of the confounding factors, psychological distress. We also found that subjects with frequent meal-related abdominal pain more often use healthcare services for bowel problems. Most likely patients who report meal-related symptoms could benefit from a multidisciplinary care approach including dietary and lifestyle advice and psychological and pharmacological therapy [60]. This could make the treatment approach more efficient by targeting the burden of the non-GI somatic symptoms and psychological distress, the poor quality of life, and the diverse GI symptom pattern all at once.

The key strength of our study is the use of a global-reaching, uniformly collected dataset. The general population sampling was done with different quotas, allowing demographically balanced and population-representative samples with regard to age, gender, and education level. The initial epidemiology questionnaire study was collaboratively designed by a team of international DGBI experts, generating a very rich set of information focusing on the evaluation of all DGBI diagnoses using the latest diagnostic Rome IV criteria. All questionnaires were translated using a uniform translation method with cultural adaptations and linguistic validations. Quality checks were included in the online survey and the subsequent data processing to ensure that inconsistent and non-attentive responders could be eliminated from the analyses. Nevertheless, our study has several limitations. The first limitation is the cross-sectional nature of the data. It has been demonstrated that the severity of symptoms that patients with DGBI experience varies over time and that they can potentially shift between DGBI diagnoses depending on their symptom profile [61]. In addition, despite the fact that we identified and excluded subjects with common doctor-diagnosed organic GI diseases (such as celiac disease, inflammatory bowel disease, cancer anywhere in the GI tract, peptic ulcer, and diverticulitis) from DGBI diagnoses, we could potentially have missed less common organic GI conditions that could explain the symptom profile. Furthermore, since the survey was conducted anonymously, we could not invite subjects to verify their clinical history or do medical tests to exclude alternative causes of the GI symptoms in our study cohort. This specific analysis assessing meal-related abdominal pain also included a specific limitation. The analysis and the categorization of subjects regarding the frequency of meal-related abdominal pain were based on a single item in the survey. It could have been relevant to assess not only the frequency of the GI symptom, but in addition also the severity, burden and/or interference with daily life, and other meal-related symptoms apart from abdominal pain.

## Conclusion

In conclusion, this study demonstrates that a substantial proportion of the worldwide population experiences frequent meal-related abdominal pain. Identifying these subjects in the general population is important to assess the magnitude of this problem. However, identifying them in clinical practice could also be of importance. This could allow clinicians to propose a management strategy that focuses more on meal-related issues, potentially involving the help of trained dieticians. In order to improve the management of these subjects, more studies investigating meal-related GI symptoms (beyond abdominal pain) are needed. This will help us to understand the complete symptom profile of this specific subgroup and enhance our understanding of the underlying pathophysiological mechanisms.

## Supplementary Information

**Additional file 1: Figure S1.** The frequency of having other GI symptoms is associated with having meal-related abdominal pain more frequently (Unadjusted multivariable regression model). Mixed ordinal regression models with frequency of meal-related pain (11-item scale, 0-100%) as outcome indicated that having meal-related abdominal pain more frequently was associated with having other GI symptoms more frequently. The frequency of all other GI symptoms questioned in the Adult Diagnostic Rome IV questionnaire were used as an independent variable for the separate mixed ordinal regression models. Country was included as random intercept effect to account for variability among countries. OR>1 correspond to higher odds of having meal-related abdominal pain more frequently. **Table S1.** Base model of the mixed ordinal regression*. **Table S2.** The degree of occurrence of other GI symptoms in subjects grouped by meal-related abdominal pain occurrence*. **Table S3.** Mixed linear regression unadjusted*.

## Data Availability

The data that support the findings of this study are available from Rome Foundation Global Epidemiology Study, but restrictions apply to the availability of these data, which were used under license for the current study, and so are not publicly available. Data are however available from the authors upon reasonable request and with permission of the Rome Foundation Global Epidemiology Study.

## Abbreviations

95% CIs: 95% confidence intervals
ANOVA: Analysis of variance
BMI: Body mass index
DGBI: Disorder(s) of gut-brain interaction
FGIDs: Functional gastrointestinal disorders
FODMAPs: Fermentable oligo-, di-, monosaccharides, and polyols
GI: Gastrointestinal
IBS: Irritable bowel syndrome
IgE: Immunoglobulin E
OR: Odds ratio
PHQ: Patient health questionnaire
PROMIS-10: Patient-Reported Outcomes Measurement Information System-10
