# Supplementary material for: Global prevalence and burden of meal-related abdominal pain
Source: BMC Med. 2022 Feb 17;20:71. doi: 10.1186/s12916-022-02259-7 (PMC8851773; doi:10.1186/s12916-022-02259-7)
Supplement: Supplementary file 1 — Additional file 1: Figure S1. The frequency of having other GI symptoms is associated with having meal-related abdominal pain more frequently (Unadjusted multivariable regression model). Mixed ordinal regression models with frequency of meal-related pain (11-item scale, 0-100%) as outcome indicated that having meal-related abdominal pain more frequently was associated with having other GI symptoms more frequently. The frequency of all other GI symptoms questioned in the Adult Diagnostic Rome IV questionnaire were used as an independent variable for the separate mixed ordinal regression models. Country was included as random intercept effect to account for variability among countries. OR>1 correspond to higher odds of having meal-related abdominal pain more frequently. Table S1. Base model of the mixed ordinal regression*. Table S2. The degree of occurrence of other GI symptoms in subjects grouped by meal-related abdominal pain occurrence*. Table S3. Mixed linear regression unadjusted*. [file 12916_2022_2259_MOESM1_ESM.docx]

**SUPPLEMENTARY FIGURE AND TABLES**

**Supplementary table 1. Base model of the mixed ordinal regression***

| **Variable** | **Unadjusted OR**  **(95% CI)** | **Adjusted OR**  **(95% CI)** |
| --- | --- | --- |
| Age (10 years) | **0.88 (0.87, 0.89)** | **0.91 (0.89, 0.92)** |
| Female gender | **1.05 (1.00, 1.09)** | **0.95 (0.91, 1.00)** |
| BMI (kg/m^2^) | 1.00 (0.99, 1.00) | 1.00 (0.93, 1.02) |
| Education (4 years) | 0.99 (0.97, 1.01) | 1.01 (0.99, 1.03) |
| Psychological Distress (0-12 points) | **1.14 (1.13, 1.15)** | **1.13 (1.12, 1.14)** |

*For the multivariable analysis, this base mixed ordinal regression model used. The frequency of meal-related abdominal pain (11-item scale) was the outcome. Per model, the frequency of a separate other GI symptoms was added to this base model with the controlling factors; demographical variables (age, gender, education, BMI), and psychological distress. The variables ‘age’ and ‘education’ were entered in 10-year and 4-year steps, respectively. Phycological distressed (measured with the PHQ-4 was entered as 13-item scale. Bold OR had a significant (p<0.05) main effect on the frequency of meal-related abdominal pain.

**Supplementary table 2. The degree of occurrence of other GI symptoms in subjects grouped by meal-related abdominal pain occurrence. ***

| ***Other GI symptoms***  *(scored on 9-item and 11-item scales)* | **Occurrence of meal-related abdominal pain** | | |
| --- | --- | --- | --- |
|  | **No**  **(n=9,756)** | **Occasional**  **(n=12,377)** | **Frequent**  **(n=5,932)** |
| ***9-item scale*** |  |  |  |
| Sensation of a lump in the throat | 1.07 (1.04, 1.12) | 1.65 (1.62, 1.68) | 2.12 (2.06, 2.17) |
| Retrosternal chest pain | 0.85 (0.82, 0.87) | 1.29 (1.27, 1.32) | 1.82 (1.77, 1.87) |
| Heartburn | 1.38 (1.35, 1.42) | 1.95 (1.92, 1.98) | 2.59 (2.53, 2.65) |
| Dysphagia | 0.64 (0.62, 0.66) | 1.18 (1.16, 1.21) | 1.77 (1.72, 1.92) |
| Postprandial fullness | 1.19 (1.16, 1.22) | 2.09 (2.06, 2.12) | 2.85 (2.79, 2.91) |
| Early satiety | 1.03 (1.00, 1.06) | 1.79 (1.76, 1.82) | 2.51 (2.45, 2.56) |
| Epigastric pain and burning | 0.38 (0.37, 0.40) | 1.09 (1.06, 1.11) | 1.94 (1.89, 1.99) |
| Nausea | 0.51 (0.49, 0.53) | 1.06 (1.04, 1.09) | 1.68 (1.62, 1.73) |
| Vomiting | 0.33 (0.31, 0.34) | 0.61 (0.59, 0.63) | 0.98 (0.94, 1.02) |
| Regurgitation | 0.47 (0.45, 0.49) | 0.94 (0.92, 0.96) | 1.50 (1.45, 1.55) |
| Belching | 0.35 (0.33, 0.37) | 0.94 (0.91, 0.96) | 1.46 (1.41, 1.51) |
| General abdominal pain | 1.89 (1.87, 1.92) | 2.32 (2.30, 2.34) | 3.39 (3.34, 3.43) |
| Bloating or abdominal distention | 1.82 (1.79, 1.86) | 2.58 (2.54, 2.61) | 3.59 (3.53, 3.65) |
| Biliary pain | 0.95 (0.92, 0.97) | 1.67 (1.64, 1.69) | 2.56 (2.50, 2.61) |
| Accidental leakage of stool | 0.19 (1.17, 0.20) | 0.38 (0.37, 0.40) | 0.65 (0.61, 0.68) |
| Aching, pain or pressure in rectum not associated with bowel movement | 0.50 (0.48, 0.52) | 0.99 (0.96, 1.01) | 1.60 (1.55, 1.65) |
| ***11-item scale*** |  |  |  |
| Hard/lumpy stool | 1.70 (1.65, 1.74) | 2.35 (2.31, 2.39) | 3.30 (3.23, 3.37) |
| <3 stools per week (without laxative medication or enema) | 1.35 (1.30, 1.41) | 2.03 (1.98, 2.08) | 2.94 (2.85, 3.02) |
| Stool straining | 2.12 (2.07, 2.16) | 2.90 (2.85, 2.94) | 4.21 (4.13, 4.29) |
| Feeling of incomplete emptying | 2.18 (2.13, 2.23) | 2.48 (2.43, 2.52) | 4.66 (4.58, 4.73) |
| Sensation of blocked stool | 1.55 (1.50, 1.59) | 2.48 (2.43, 2.52) | 3.82 (3.74, 3.89) |
| Manual help for stool emptying | 0.51 (0.48, 0.54) | 0.96 (0.93, 1.00) | 1.64 (1.57, 1.71) |
| Mushy/watery stool | 1.42 (1.38, 1.46) | 2.13 (2.09, 2.17) | 3.30 (3.23, 3.37) |
| Meal-related mushy/watery stool | 1.88 (1.82, 1.95) | 2.56 (2.52, 2.61) | 4.25 (4.17, 4.33) |
| Bowel movement urgency | 1.44 (1.40, 1.48) | 2.26 (2.22, 2.30) | 3.52 (3.44, 3.59) |

*The mean frequency of GI symptoms measures with the Adult Diagnostic Rome IV questionnaire were compared between the three groups of meal-related abdominal pain. A mean score 2 on the 9-item scale (0: never, 8: multiple times per day or all the time) corresponds to 1 day a month (in the last 3 months) and a score of 2 on the 11-item scale (0: 0% of the time, 10: 100% of the time) corresponds to 20% of the time (in the last 3 months).

‘No’: abdominal pain 0% of the time meal-related; ‘occasional’: abdominal pain 10-40% of the time meal-related; ‘Frequent’: abdominal pain ≥ 50% of the time meal-related; Data are presented as percentage (95% confidence interval).

**Supplementary table 3. Mixed linear regression unadjusted***

| **Outcome** | **Psychological distress** | **Non-GI somatic symptoms** | **Physical**  **Quality of Life** | **Mental**  **Quality of Life** |
| --- | --- | --- | --- | --- |
| Predictors | Estimates (95% CI) | | | |
| Intercept | 3.04  (2.82, 3.26) | 6.20  (6.03, 6.37) | 14.17  (13.98, 14.36) | 13.18  (12.82, 13.55) |
| Meal-related abdominal pain | 0.24  (0.22, 0.25) | 0.35  (0.33, 0.36) | -0.22  (-0.23, -0.21) | -0.17  (-0.19, -0.16) |

*In the unadjusted models, the frequency of meal-related abdominal pain had a significant (p<0.05) main effect on all different outcomes.

**
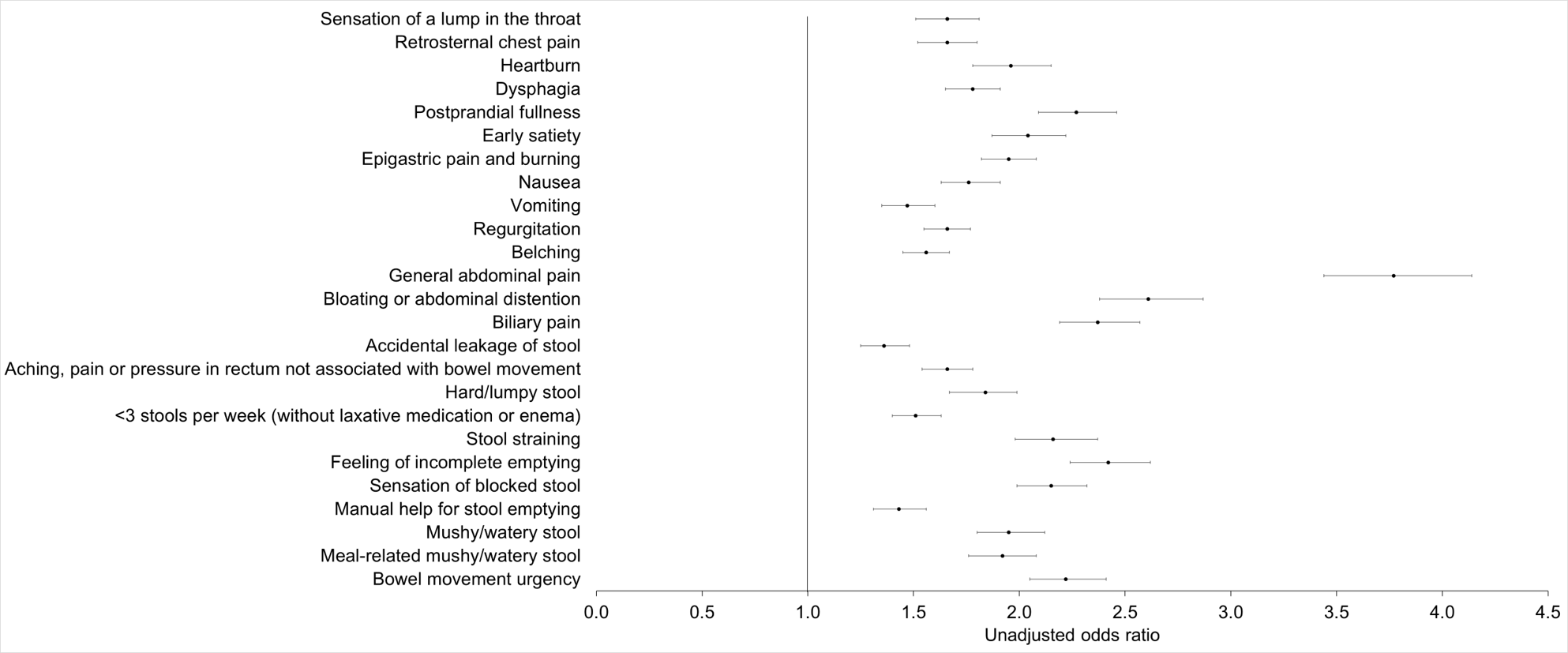
**

**Supplementary figure 1. The frequency of having other GI symptoms is associated with having meal-related abdominal pain more frequently (Unadjusted multivariable regression model)**

Mixed ordinal regression models with frequency of meal-related pain (11-item scale, 0-100%) as outcome indicated that having meal-related abdominal pain more frequently was associated with having other GI symptoms more frequently. The frequency of all other GI symptoms questioned in the Adult Diagnostic Rome IV questionnaire were used as an independent variable for the separate mixed ordinal regression models. Country was included as random intercept effect to account for variability among countries. OR>1 correspond to higher odds of having meal-related abdominal pain more frequently.
